# Validation of a novel point of care testing device for sickle cell disease

**DOI:** 10.1186/s12916-015-0473-6

**Published:** 2015-09-16

**Authors:** Julie Kanter, Marilyn J. Telen, Carolyn Hoppe, Christopher L. Roberts, Jason S. Kim, Xiaoxi Yang

**Affiliations:** Pediatrics, Medical University of South Carolina, Charleston, SC USA; Division of Hematology, Department of Medicine, Duke University Medical Center, Durham, NC 27710 USA; Children’s Hospital Oakland, Oakland, CA 94609 USA; BioMedomics, Inc., Durham, NC USA; Sickle Cell Research, Medical University of South Carolina, 135 Rutledge Avenue, MSC 558, Charleston, SC 29425 USA

**Keywords:** Sickle cell, Point of care, Diagnostics, Immunoassay

## Abstract

**Background:**

Sickle cell disease is one of the most common inherited blood disorders. Universal screening and early intervention have significantly helped to reduce childhood mortality in high-resource countries. However, persons living in low-resource settings are often not diagnosed until late childhood when they present with clinical symptoms. In addition, confirmation of disease in affected individuals in the urgent care setting is limited in both high- and low-resource areas, often leading to delay in treatment. All of the current diagnostic methods rely on advanced laboratory systems and are often prohibitively expensive and time-consuming in low-resource settings. To address this need, the Sickle SCAN™ test has been developed to diagnose sickle cell disease and sickle cell trait at the point of care without electricity or advanced equipment.

**Methods:**

This study was conducted to evaluate and validate the diagnostic accuracy of the Sickle SCAN™ test, a novel point of care test for sickle cell disease. Thus, we describe the laboratory testing and clinical validation of the Sickle SCAN™ test in individuals >1 year of age using capillary blood. The Sickle SCAN™ test was created using advanced, qualitative lateral flow technology using capillary blood to identify the presence of hemoglobin A, S, and C allowing for detection of results with the naked eye.

**Results:**

Laboratory testing using venous blood demonstrated 99 % sensitivity and 99 % specificity for the diagnosis of HbSS, HbAS, HbSC, HbAC, and HbAA. Seventy-one subjects underwent capillary blood sampling at the point of care for further validation. This test detected the correct A, S, and C presence with an overall diagnostic accuracy of 99 % at the bedside.

**Conclusion:**

The Sickle SCAN™ test has the potential to significantly impact the diagnosis and treatment for sickle cell disease worldwide as well as enhance genetic counseling at the point of care. Further validation testing will be conducted in newborns in resource-poor settings in upcoming studies.

**Electronic supplementary material:**

The online version of this article (doi:10.1186/s12916-015-0473-6) contains supplementary material, which is available to authorized users.

## Background

Sickle cell disease (SCD) is one of the most common inherited blood disorders in the world. SCD is caused by the inheritance of two copies of the gene encoding hemoglobin S, a protein that results from a missense mutation in the β-globin subunit of hemoglobin A, or the co-inheritance of the gene for hemoglobin S and another abnormal or nonfunctional hemoglobin gene [1]. Resulting erythrocytes are both unstable, leading to excessive hemolysis, and abnormal, leading to inflammation and vascular occlusion. Patients with SCD have a multitude of complications, including pain, infections, stroke, acute chest syndrome (sickling and occlusion within the pulmonary vasculature), and multi-organ damage [2]. However, improvements in early diagnosis and enhanced preventive care treatment in high-resource countries has led to a dramatic improvement in childhood survival and increase in average lifespan of affected individuals [3].

Many of the current improvements in treatment of persons with SCD are the direct result of newborn screening, which became universal in the United States 15 years ago [4]. Early diagnosis allows for the initiation of prophylactic antibiotics as well as education of affected families on the importance of immediate evaluation for children with symptoms of complications. However, many low-resource countries in which SCD is far more prevalent remain unable to provide universal newborn screening, and affected children may not be diagnosed until they present with symptoms. In addition to the financial limitations of newborn screening, some countries with relatively high incidences of SCD are also limited by governments that may not recognize or agree with the importance or utility of early testing.

Even in the United States, there remain many barriers to care for persons with SCD. Pain is the hallmark symptom of SCD and is the primary reason for which affected patients seek care [5]. Adult persons with SCD often rely on urgent care services due to a paucity of trained and available specialty providers. In many community hospitals and rural areas, physicians may not have the capability to diagnose affected individuals or confirm disease in patients seeking urgent care. Even when testing is available, results are often delayed and not available until after patients have left the hospital setting. This inability to validate disease status may lead to delay in treatment for affected patients or use of treatment in patients not actually affected by SCD.

The validation of a simple, rapid, electricity-free bedside test for SCD could transform clinical care for affected persons in both low-income developing countries and urgent care settings. The goal of this study was to test the diagnostic accuracy, including the sensitivity, specificity, and limit of detection (LoD) of this novel testing device (Sickle SCAN™) in the laboratory, and confirm the feasibility and validity of the test when used at the bedside. It was important that the results demonstrate that the new device is easy to use, can be performed with capillary blood (finger stick), and that results are easily viewed at the point of care (POC).

## Methods

### Design of testing device

The Sickle SCAN™ test was designed as a rapid, highly sensitive test. It was created using advanced, qualitative lateral flow technology to identify sickle cell disorders of hemoglobin A, S, and C allowing for detection of results with the naked eye in the POC setting. The test was specifically developed to allow for the confirmation of sickle cell trait (HbAS) and SCD in persons with HbSS, HbSC, HbSβ^0^, and HbSβ^+^ genotypes.

**Fig. 1:**
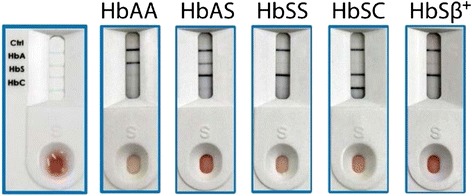
Sickle SCAN™ test performance

The test requires 5 μL of blood added to a provided buffer-loaded module designed to release hemoglobin by lysing erythrocytes. The resulting hemolyzed solution is dropped onto the sample inlet of the Sickle SCAN™ cartridge. The treated sample flows through the test cartridge in order to interact with antibody-conjugated colorimetric detector nanoparticles and travel to the capture zones (identified by lines on the device). A total of four detection lines are possible, including hemoglobin variants A, S, C, and a control line (which confirms the test is functioning). Samples containing two hemoglobin variants (such as compound heterozygotes) will have both hemoglobin variants detected (Fig. 1).

### Test principle

The Sickle SCAN™ assay employs the sandwich format chromatographic immunoassay approach for the qualitative measurement of human HbA, HbS, and HbC in whole blood samples. A mouse monoclonal antibody (MsxHb-15001, BioMedomics, Inc., Durham, NC, USA) against the C-terminus of human hemoglobin α-chain is used as the detection antibody. This detection antibody is conjugated to blue colored nanoparticles (BMBB-32-14001, 300 nm, BioMedomics, Inc.). Three polyclonal antibodies against the initial N-terminal amino acid sequence (BioMedomics, Inc.) of human sickle cell hemoglobin (HbS), human hemoglobin C (HbC), and adult normal hemoglobin (HbA) are used as capture antibodies on test lines. A separate goat anti-mouse IgG (H&L) antibody (GtxMu-003-E, ImmunoReagents, Inc., Raleigh, NC, USA) is used as the capture antibody to form the control line.

As the test sample diffuses through the absorbent test strip, the antibody-conjugated colorimetric detector nanoparticles bind to the hemoglobin in the specimen, forming an antibody-antigen complex. The specimen then migrates across a membrane toward three test lines containing HbA, HbS, and HbC antibodies to selectively detect the presence of each Hb. The specific complex with each Hb is captured at the test line with the corresponding antibody and produces a blue colored band. Excess conjugate will flow past the test lines and be captured on the control line. Therefore, to serve as a procedural control, a colored band will always appear at the control line region if the proper volume of sample has been added and membrane wicking has occurred. Once the test has run and the control line appears, the presence of any of those test lines indicates that the respective hemoglobin is present in the blood. This allows us to identify persons with hemoglobin A, sickle trait (AS), HbSS, and HbSC disease. However, the LoD of hemoglobin A (40 %) is higher than that of hemoglobin S (2 %). This was an intentional design in order to ensure persons with HbSβ^+^ were identified with sickle cell disease (although they are seen with this test as HbSS) and still differentiate sickle cell trait (HbAS). As a result of this distinction the intensity of the test lines does not correlate in this initial Sickle SCAN™ test to the quantity of the specific hemoglobin. In accordance, the test is not designed to identify less common hemoglobin variants (such as hemoglobin Constant Spring, hemoglobin O-Arab, or others). Thus, results indicating hemoglobin A alone is not diagnostic and further testing should be considered in the context of an individual’s ethnic background.

### Design of testing device

In an effort to improve rapid test specificities and sensitivities, this test was developed using advanced, qualitative lateral flow technology and detection requiring only the naked eye. This Sickle SCAN™ test was created to identify sickle cell disorders of hemoglobin A, S, and C.

**Fig. 2:**
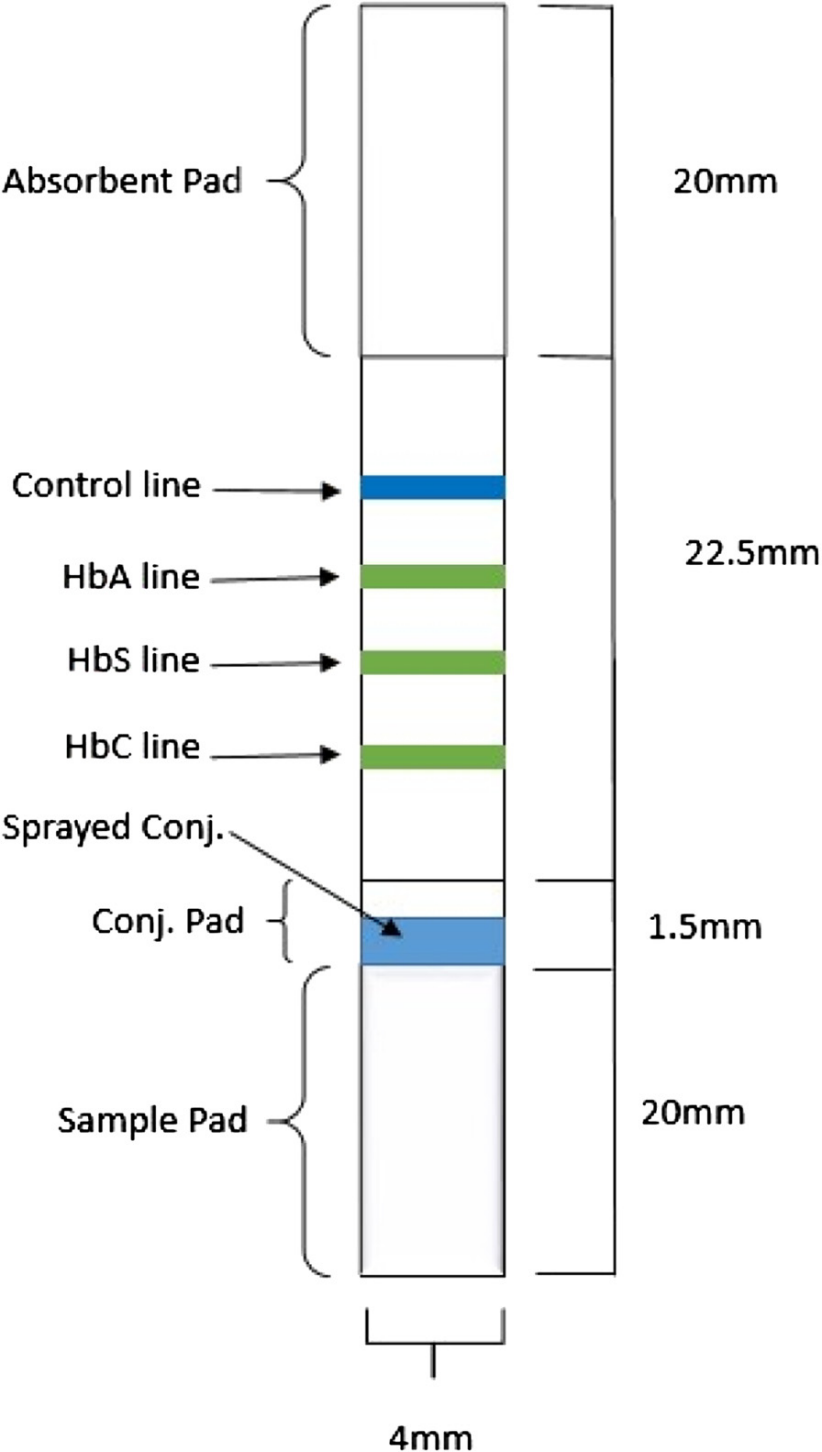
Schematic illustration of the design of Sickle SCAN™ strip with absorbent pad, control line, HbA line, HbS line, HbC line, conjugate pad, sprayed conjugates, and sample pad

**Fig. 3:**
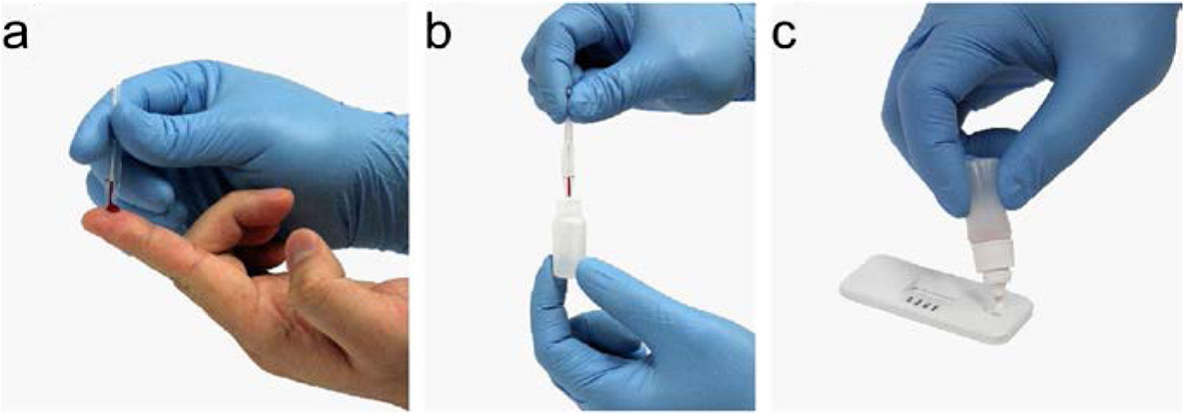
Sickle SCAN™ test procedure. **a** Five microliters of finger stick sample is taken using the capillary sampler. **b** The sample is then mixed with the pretreatment buffer. **c** Five drops of the diluted sample are then dispensed onto the test well. Figure reproduced with permission from BioMedomics

Sickle SCAN™ is a qualitative lateral flow immunoassay that tests for the presence of hemoglobin A, S, and C (Fig. 2). The kit includes the immunoassay, capillary sampler, and pretreatment buffer. Five microliters of sample is taken using the capillary sampler (Fig. 3a) and then diluted in the pretreatment buffer (Fig. 3b). Once diluted, five drops of the sample are dispensed onto the immunoassay and the test is read after 2 minutes (Fig. 3c).

### *Ex vivo* laboratory testing methods

Patient samples were obtained from venipuncture performed at the Medical University of South Carolina (Charleston, SC, USA), Duke University (Durham, NC, USA), and Children’s Hospital Oakland (Oakland, CA, USA). The collection and use of these samples for test development were approved by the local institutional review boards (IRBs) in each institution above. Patients were recruited from the regular SCD clinic populations. Samples were collected in EDTA and kept at room temperature for shipment to BioMedomics, Inc. Testing occurred within 4 weeks of sample receipt. Those who had received a blood transfusion within the last 60 days were excluded from analysis.

Five microliters of venous sample (taken from the EDTA-stored samples) were mixed in 1 ml of hemoglobin solubility buffer (used to lyse erythrocytes) designed specifically for this device. The sample was mixed by inversion for 20 seconds and then five drops (using the designated dropper) of hemolyzed solution were dropped onto the inlet to the Sickle SCAN™ testing platform. Ten minutes elapsed prior to quantification of the test line color intensity. Data collection and reference standards were planned as per the test principle prior to the index test.

The initial quantification of the Sickle SCAN™ test line color intensity was accomplished by removing the assay strip from the device cartridge and scanning it using a portable flatbed scanner (CanoScan LiDE210, Canon, Melville, NY, USA). The image was then analyzed using a custom-coded algorithm (MATLAB, MathWorks, Cambridge, UK) to determine the color intensity of the test lines. The quantitative analysis of the test line intensities was determined by the RGB color model values of the image at the test line positions. The software automatically determined the test line positions by searching for the control line, present on all tests, and measuring a set distance to the next test line position. An intensity cutoff was determined to distinguish between positive and negative results for each line. Sickle SCAN™ was compared to either hemoglobin electrophoresis (HYDRASYS acid assay, Sebia, Norcross, GA, USA) or high performance liquid chromatography (HPLC) for each sample using standard guidelines. Confirmatory testing using the above techniques was performed individually at the designated institutions. The results of these confirmatory tests are thus described interchangeably as the gold standard diagnostics. Of note, a scanner is not required for the POC test but was used here for confirmation during analysis.

### Limit of detection (LoD)

The LoD is the minimum percent of a specific Hb, that is, HbA, in which the indicator can be read. The percent Hb is the percentage of the specific hemoglobin out of the total Hb concentration. The LoD for the HbA indicator was tested in samples ranging from 0–60 % HbA in increments of 10 %. The LoD for the HbS was tested with samples ranging from 0–40 % HbA in varying increments, and the LoD for the HbC indicator was tested with samples containing 0–10 % HbC in increments of 2 %. The samples for the HbA, HbS, and HbC LoD studies were prepared, respectively, by mixing HbA, HbS, and HbA + C standards with either HbS or HbA standards to create the desired specific Hb percentages. Each sample was loaded onto the Sickle SCAN™ assay and allowed to run for 10 minutes.

### Interfering factors

Whole blood samples with BSA at concentrations of 0–100 mg/mL, penicillin at concentrations of 0–500 μg/mL, hydroxyurea at concentrations of 0–75 μg/mL, bilirubin at concentrations of 0–2.5 μg/mL, and cholesterol at concentrations of 0–4 mg/mL were tested in this interference study.

### Capillary testing methods

All patients signed written consent to participate in testing by finger stick at the POC. For pediatric patients under 18 years of age, informed consent to participate in the study was obtained from their parent or guardian. IRB approval was obtained from the Medical University of South Carolina (MUSC) to conduct a pilot trial using this POC test device in patients with SCD and their family members followed at the MUSC SCD clinic. All SCD providers in the clinic were trained in reading the testing device prior to the initiation of testing.

Subjects of all ages were included in the trial. According to the approved IRB protocol, all included patients with SCD had to have a current HPLC hemoglobin variant result. In most infants between 6–12 months of age, HPLC is not assessed routinely after the initial confirmatory assessment. Thus, although the LoD for both HbS and HbC (1 % and 2 %, respectively) discussed above in the *ex vivo* study demonstrates that the test will likely be useful in newborn children, they were not included in this validation study and a follow-up study in neonates will be undertaken. Those who had received a blood transfusion within the last 60 days or whose hemoglobin genotype was not previously known were excluded. Patients receiving hydroxyurea therapy were included. At the time of consent, 71 patients met inclusion and exclusion criteria based on their history and were consented to undergo testing. Blood was obtained from the finger pad using a standard lancet for capillary testing. Five microliters of blood was collected using the capillary sampler provided in the testing kit and added to the buffered loaded module. After inverting the module × 3, five drops of hemolyzed sample were dropped onto the inlet of the Sickle SCAN™ cartridge. Results were read within 120 seconds. The Sickle SCAN™ test result was determined after visual inspection sequentially by two independent providers who determined the results separately without reference to one another. Due to timing, the initial provider read the test at 2 minutes and the second provider read the test between 4–10 minutes after sample had been added. In affected patients, the first provider had knowledge of the patient’s genotype prior to testing. However, the second provider was always blind to the sample being tested. On average, all testers reviewed each sample within 5 minutes of testing.

## Results

### *Ex vivo* laboratory testing

#### Ex vivo *laboratory testing results*

Sickle SCAN™ results were compared to gold standard testing by either hemoglobin electrophoresis (HYDRASYS acid assay, Sebia) or HPLC (Finnigan Surveyor, Thermo Electron Corporation, Waltham, MA, USA, and VARIANT II TURBO, BioRad, Hercules, CA, USA) using standard guidelines at the institutions where samples were collected. Patient samples (n = 137) were collected and measured in duplicate (on the same sample) using Sickle SCAN™ and compared to the gold standard diagnostic test obtained at the institution where the sample initiated (Table 1).

**Table 1:** Sickle SCAN™ performance compared to genotypes identified by gold standard diagnostic testing

Sickle scan test result						
	SS	AS	SC	AC	AA	Total
HbSS	42	0	0	0	0	42
HbAS	0	24	0	0	0	24
HbSC	0	0	37	0	0	37
HbAC	0	0	0	4	0	4
HbAA	0	0	0	0	30	30
Total	42	24	37	4	30	137
Specificity	>99 %	>99 %	>99 %	>99 %	>99 %	>99 %
Sensitivity	>99 %	>99 %	>99 %	>99 %	>99 %	>99 %

#### Limit of detection (LoD) results

The Sickle SCAN™ LoD for hemoglobin A, S, and C was determined to be 40 %, 1 %, and 2 %, respectively (Additional file 1: Tables S1–S3). The LoD for HbA was set higher than for HbS and HbC both to allow: a) patients with HbSβ^+^ who usually have up to 25 % HbA to be diagnosed as HbS using Sickle SCAN™, and to be differentiated from those with HbAS who have >40 % HbA and will be visualized as ‘AS’; and b) to ensure the LoD for HbS and HbC were small enough to make this test useful in the neonatal period.

#### Interfering factors

Results demonstrated that Sickle SCAN™ had no interference by the following substances at the concentrations indicated: protein (BSA) 100 mg/mL; penicillin 250 μg/mL; hydroxyurea 75 μg/mL; bilirubin 2.5 mg/mL; and cholesterol 4 mg/mL (Additional file 1: Table S4–S8).

#### Reading time factor

The optimal time to interpret the results was determined using one clinical AA sample and one clinical SC sample. These tests were run together and scanned using a portable flatbed scanner (CanoScan LiDE210, Canon) similar to the method used for the *ex vivo* laboratory testing. The tests were scanned for 5 minutes through 24 hours after the samples were loaded and analyzed using a custom-coded algorithm (MATLAB, MathWorks). The quantitative analysis of the test line intensities was determined by the RGB color model values of the image at the test line positions (shown in Additional file 1: Table S9). To read the tests under 5 minutes a separate study was conducted with eight clinical samples (two AA, two AS, two SC, and two SS) read by three individuals that were not provided with any identifying information on the samples. Their interpretation of the results were recorded and compared to the diagnosis of each clinical sample (Additional file 1: Table S10). The combined results from both of these reading time studies suggest that the Sickle SCAN™ can be read between 2 minutes and 24 hours after the sample has been loaded.

### Capillary testing at the point of care (POC)

Seventy-one subjects with HbAA, HbAS, HbAC, HbSS, HbSβ^+^, HbSD, or HbSC were included. Patients ranged in age from 5 weeks to 72 years. Included in this sample were 21 children (age <21 years, mean age of 13.5 years) and 50 adults (>22 years. Patients were recruited and tested from November 2014 to June 2015. There were no adverse events from the capillary sampling. Sample results are shown below with confirmed genotypes (Table 2). There was 100 % concordance among providers interpreting the results.

**Table 2:** Sickle SCAN™ results compared to known genotypes

Number of samples	Sickle SCAN™	Genotype
23	S	20 confirmed HbSS
		2 confirmed HbSβ^0^
		1 confirmed HbSβ^a,b^
14	SC	14 confirmed HbSC
21	AS	17 confirmed or previously known HbAS (trait)
		3 confirmed HbSS with recent transfusion^b^
		1 confirmed HbSD^c^
3	AC	3 persons with known HbAC
8	A	8 persons known without HbC or HbS^d^
2	C	3 confirmed with HbCC

^a^As per the purposeful test design, persons with HbSβ^+^ appear as HbSS using this test. This sample contained 24 % hemoglobin A; ^b^persons with HbSS that were found to have been transfused based on electrophoresis and further investigation after testing; ^c^erroneous result of HbAS in person with HbSD; ^d^persons noted to have HbA were known β-thalassemia carriers in two cases. Definitive electrophoresis was not performed in six cases but newborn screening reports were reportedly normal in all six persons

The Sickle SCAN™ test performed favorably with one false negative result in which the test read AS (sickle cell trait) in a patient with confirmed HbSD disease). In 71 subjects, this test detected the correct A, S, and C presence with an overall diagnostic accuracy of 99 % (Table 3).

**Table 3:** Sensitivity and specificity of Sickle SCAN™ compared to known patient genotypes

	Sensitivity (95 % CI)	Specificity (95 % Cl)
SCD (HbSS, HbSC, HbSβ^+^-thal, HbSβ^0^-thal)	100 % (91–100)	100 % (90–100)
Sickle trait (HbAS)	100 % (85–100)	98 % (90–100)
C trait (HbAC)	100 % (44–100)	100 % (95–100)
Normal (HbAA)	100 % (68–100)	100 % (94–100)

## Discussion and conclusions

According to the authors’ knowledge, there is no other similar SCD diagnostic device currently on the market. A variety of POC devices for the diagnosis of SCD are currently in development and testing, but the Sickle SCAN™ employs lateral flow immunoassay technology, which favors its utility for large-scale screening efforts in low-resource settings. In addition, other tests in development have not successfully been able to produce a test that can distinguish HbSS, HbAS, and HbSC, which makes this new device substantially more useful.

The Sickle SCAN™ differs from previous tests for determining the presence or absence of HbS and/or HbC in whole blood samples and offers significant advantages not realized in prior applications. For example, solubility testing methods such as SICKLEDEX® (Streck, Inc., Omaha, NE, USA) and concentrated phosphate buffer are simple and inexpensive, but do not differentiate between sickle cell disease (including HbSS, HbSβ^0^-thalassemia, HbSβ^+^-thalassemia, and HbSC) and sickle trait (HbAS). The methods are based on HbS polymerization (visible turbid suspension) in the presence of a concentrated phosphate buffer solution. All positive test results require further evaluation by hemoglobin electrophoresis or HPLC when used for patient screening. These deficiencies are overcome by this novel testing device.

In an optimized solubility testing method, instead of measuring turbidity, the characteristic blood stain formed on a paper-based assay becomes an active element [6] and the polymerized HbS is entangled by the paper fibers. The soluble hemoglobin will continue to spread on the paper and, because it is colored, the assay read out uses the red color count in the region of the polymerized hemoglobin and soluble hemoglobin. The visual signals need to be analyzed by a scanner to correlate the blood stain pattern with the concentration of HbS present. But this assay cannot accurately distinguish between individuals with HbAS (trait) and HbSC (disease) since they have similar HbS concentrations. In a person with HbSC, the presence of HbC enhances the pathogenic properties of HbS by inducing dehydration and therefore sickling at a significant level that would not take place in a person with similar levels of HbS without HbC. Here again, these deficiencies are overcome by Sickle SCAN™.

A hemolysis monitoring assay in non-electrolyte solutions [7] has been proposed to distinguish red blood cells from HbSS and HbAS individuals based on the altered properties of the RBC membrane resulting from HbS polymerization. However, an hour of incubation time, as well as a requirement for the use of tonometer and optical density measurements, make such a test difficult to be used at the POC or in low-resource areas that do not have the capacity to support these instruments.

The recent development of a cell density-based aqueous multiphase system requires a drop of whole blood, which goes into a capillary tube filled with three different polymeric aqueous solutions. After centrifugation in a small, battery-operated instrument, sickle cells are separated from normal red blood cells, based on differences in their cell density. The isolated sickle cell fraction then needs to be detected by a simple optical reader. The required use of centrifuge and optical reader challenges the simplicity of this POC test. Additionally, HbAA (normal) and HbAS (trait) have the same performance in this system and cannot be distinguished [8]. Once again these deficiencies are overcome by the Sickle SCAN™ test demonstrated here.

The innovative design for Sickle SCAN™ described above provides: 1) high specificity to identify HbS, HbC, and HbA, even in the presence of up to 30 % HbF (as in persons on hydroxyurea) or HbA_2_; 2) high sensitivity to simultaneously detect HbS, HbC, and HbA, even in anemic patients; and 3) an unprecedented capacity to differentiate SCD (homozygous HbSS, heterozygous HbSC, and HbSβ-thalassemia) from sickle cell trait (heterozygous HbAS) and normal adult hemoglobin (HbAA). Of note, it should be clarified that the ability to differentiate persons with HbAS and HbSβ^+^ using Sickle SCAN™ is subtle. Because individuals with HbSβ^+^ usually have less than 25 % HbA, they will appear as if they have HbSS disease using this test (compared to those with HbAS who have >40 % HbA). This difference also elucidates the reason for which the LoD for HbA was set at 40 % for this test. In contrast to the other aforementioned POC tests for SCD, this test does not require electricity, equipment, or a skilled nurse or physician to draw blood. In contrast, the test is easy to perform and results are read visually with the naked eye using the detection lines on the device.

Thus, the Sickle SCAN™ will be the first POC device that is able to diagnose and differentiate the most common forms of SCD and trait as well as HbC disease and trait. It is designed as a sensitive, rapid and low-cost test to be used as the primary diagnostic tool for SCD and sickle trait in sub-Saharan Africa, India, and other regions of the developing world. The company has already received CE mark approval and has commenced commercialization in these regions.

Currently, there are two noted limitations in the Sickle SCAN™ test.

1. At present, the LoD for hemoglobin A is set at 40 %. This was configured in order to capture persons with HbSβ^+^ disease (which are seen as HbS alone on the test results). However, it is important that testers do not conflate the weak HbA line seen in persons with HbAS as a low quantity hemoglobin. The degree of intensity of the line does not correlate with the quantity of hemoglobin. Thus, results demonstrating bands at HbS and HbA are consistent with sickle cell trait despite the fact that the band at S is darker than the band at A. This limitation is easily overcome with proper teaching of the test.
2. The Sickle SCAN™ test only shows hemoglobin A, S, and C, as described. Thus, the test cannot identify thalassemia carriers, a condition which can impact genetic counseling. Additionally, the test cannot identify all hemoglobin variants but instead is heavily reliant on the most common. Thus, results of hemoglobin A cannot be definitively correlated to the HbAA genotype based solely on the results of this test.

Further studies using the Sickle SCAN™ device are already in progress for both community-based sickle cell disease/sickle cell trait testing as well as for the diagnosis of SCD in the newborn period, which is of crucial importance. We believe this test will significantly enhance the ability to diagnose patients in low-resource areas quickly and easily in order to enhance preventative care treatment. The Sickle SCAN™ test has already obtained CE mark approval in Europe.

### Key points

This article describes the clinical validation for a novel point of care testing device, Sickle SCAN™ for the rapid diagnosis of sickle cell disease.

## Additional file

Additional file 1:
**Supplemental information.** (DOCX 170 kb)

## Abbreviations

BSA: Bovine serum albumin
CI: Confidence interval
Hb: Hemoglobin
HbA: Adult hemoglobin
HbC: Hemoglobin C
HbS: Sickle cell hemoglobin
HPLC: High performance liquid chromatography
IRB: Institutional review board
LoD: Limit of detection
MUSC: Medical University of South Carolina
POC: Point of care
SCD: Sickle cell disease
