# Supplementary material for: Validation of a novel point of care testing device for sickle cell disease
Source: BMC Med. 2015 Sep 16;13:225. doi: 10.1186/s12916-015-0473-6 (PMC4573998; doi:10.1186/s12916-015-0473-6)
Supplement: Additional file 1: — Supplemental information. (DOCX 170 kb) [file 12916_2015_473_MOESM1_ESM.docx]

**Supplemental Information**

*Limit of Detection testing*

To determine the limit of detection (LoD), the Sickle SCAN^TM^ was run on several sets of samples with sequentially decreasing percentages of hemoglobin A, S, and C. The samples were created using HbAC (HC-105, ACS, Inc.), HbAF (HC-103, ACS, Inc.), HbA (HO267, Sigma-Aldrich Co.), and HbS (HO392, Sigma-Aldrich Co.) standards. The samples were diluted in solubility buffer at different ratios to replicate the concentration of whole blood between 9.25-10 g/dL diluted at a 200:1 ratio in buffer. The hemoglobin concentration remained the same from sample to sample in each preparation.

*Interference testing*

To determine possible interfering factors, studies were run on the Sickle SCAN™ assay with samples containing varying amounts of protein (albumin) (30-AB81, Fitzgerald Industries International, Inc.), bilirubin (B4126, Sigma-Aldrich Co.), triglycerides (I141, Sigma-Aldrich Co.), Hydroxyurea (H8627, Sigma-Aldrich Co.), and penicillin (PENNA-100MU, Sigma-Aldrich Co.). Patients screened for possessing sickle cell disorders commonly have increased amounts of these compounds due to their symptoms or treatment. Buffers were spiked with increasing amounts of these compounds and mixed with whole blood samples with known hemoglobin concentrations at a 200:1 ratio, and run with the assay. A range of values for each possible interfering factor was determined by finding the highest concentration in a clinical setting and multiplying it by a safety factor of 1.5-2 to ensure the test would function properly under extreme conditions.

**Table S1. Summary of Results From %HbA Limit of Detection Study**

| **%A** | **A-line data** | | | **S-line data** | | |
| --- | --- | --- | --- | --- | --- | --- |
|  | **1st** | **2nd** | **3rd** | **1st** | **2nd** | **3rd** |
| 0% | / | / | / | 0.71693 | 0.70858 | 0.7342 |
| 10% | / | / | / | 0.67492 | 0.68164 | 0.67548 |
| 20% | / | / | / | 0.69127 | 0.68232 | 0.67785 |
| 30% | / | / | / | 0.71181 | 0.67098 | 0.67532 |
| 40% | / | / | / | 0.65592 | 0.64961 | 0.66395 |
| 50% | 0.36401 | 0.34828 | 0.35871 | 0.67494 | 0.65397 | 0.65932 |
| 60% | 0.41728 | 0.41756 | 0.34192 | 0.64826 | 0.65633 | 0.64005 |

**Table S2. Summary of Results From %HbS Limit of Detection Study**

| **%HbS** | **A-line data** | | | **S-line data** | | |
| --- | --- | --- | --- | --- | --- | --- |
|  | **1st** | **2nd** | **3rd** | **1st** | **2nd** | **3rd** |
| 0% | 0.54817 | 0.51549 | 0.51111 | / | / | / |
| 1% | 0.5703 | 0.50051 | 0.48046 | 0.38568 | 0.3644 | 0.35224 |
| 2% | 0.57099 | 0.55216 | 0.48875 | 0.40197 | 0.40851 | 0.38705 |
| 3% | 0.5126 | 0.55139 | 0.58325 | 0.40657 | 0.42517 | 0.44242 |
| 4% | 0.49853 | 0.5086 | 0.54075 | 0.41037 | 0.41205 | 0.41899 |
| 5% | 0.53786 | 0.51598 | 0.50747 | 0.42046 | 0.41254 | 0.40023 |
| 10% | 0.5035 | 0.48947 | 0.5355 | 0.48447 | 0.5071 | 0.5041 |
| 15% | 0.48498 | 0.49823 | 0.47824 | 0.53086 | 0.53229 | 0.51348 |
| 20% | 0.47757 | 0.47982 | 0.48148 | 0.62869 | 0.58866 | 0.61038 |

**Table S3. Summary of Results From %HbC Limit of Detection Study**

| **%C** | **A-line data** | | | **C-line data** | | |
| --- | --- | --- | --- | --- | --- | --- |
|  | **1st** | **2nd** | **3rd** | **1st** | **2nd** | **3rd** |
| 0% | 0.52805 | 0.52032 | 0.52397 | / | / | / |
| 2% | 0.52278 | 0.56883 | 0.53002 | 0.40828 | 0.41837 | 0.41647 |
| 4% | 0.53345 | 0.50869 | 0.5471 | 0.46851 | 0.4656 | 0.48754 |
| 6% | 0.51791 | 0.50322 | 0.53329 | 0.49969 | 0.47664 | 0.51444 |
| 8% | 0.52143 | 0.51669 | 0.53016 | 0.53898 | 0.57651 | 0.63921 |
| 10% | 0.46825 | 0.46681 | 0.48208 | 0.52148 | 0.53247 | 0.52064 |

**Table S4. Summary of Results From protein (BSA) Interference Study**

| **BSA in whole blood, mg/mL** | **Sample** | **A-line data** | **S-line data** | **C-line data** |
| --- | --- | --- | --- | --- |
|  |  |  |  |  |
| **0** | SC | / | 0.57337 | 0.66779 |
|  | AA | 0.4433 | / | / |
| **0** | SC | / | 0.60226 | 0.63683 |
|  | AA | 0.40768 | / | / |
| **25** | SC | / | 0.59151 | 0.62507 |
|  | AA | 0.38844 | / | / |
| **50** | SC | / | 0.59647 | 0.67759 |
|  | AA | 0.41399 | / | / |
| **100** | SC | / | 0.59089 | 0.66834 |
|  | AA | 0.43664 | / | / |

**Table S5 Summary of Results From Penicillin Interference Study**

| **Penicillin in whole blood, µg/mL** | **Sample** | **A-line data** | **S-line data** | **C-line data** |
| --- | --- | --- | --- | --- |
| **0** | SC | / | 0.59018 | 0.6295 |
|  | AA | 0.45929 | / | / |
| **10** | SC | / | 0.60131 | 0.66399 |
|  | AA | 0.51578 | / | / |
| **50** | SC | / | 0.58787 | 0.71823 |
|  | AA | 0.48877 | / | / |
| **100** | SC | / | 0.58534 | 0.67105 |
|  | AA | 0.48591 | / | / |
| **250** | SC | / | 0.60789 | 0.68495 |
|  | AA | 0.46093 | / | / |
| **500** | SC | / | 0.5898 | 0.65463 |
|  | AA | 0.48436 | 0.33796 | / |

**Table S6. Summary of Results From Hydroxyurea Interference Study**

| **Hydroxyurea Concentration in Whole Blood, µg/mL** | **Sample** | **A-line data** | **S-line data** | **C-line data** |
| --- | --- | --- | --- | --- |
| **0** | SC | / | 0.63054 | 0.68068 |
|  | AA | 0.3918 | / | / |
| **10** | SC | / | 0.63287 | 0.71139 |
|  | AA | 0.44826 | / | / |
| **25** | SC | / | 0.62044 | 0.70417 |
|  | AA | 0.45482 | / | / |
| **50** | SC | / | 0.61659 | 0.7555 |
|  | AA | 0.40272 | / | / |
| **75** | SC | / | 0.59477 | 0.65928 |
|  | AA | 0.40549 | / | / |

**Table S7. Summary of Results From Bilirubin Interference Study**

| **Bilirubin Concentration in Whole Blood, µg/mL** | **Sample** | **A-line data** | **S-line data** | **C-line data** |
| --- | --- | --- | --- | --- |
| **0** | SC | / | 0.52295 | 0.63311 |
|  | AA | 0.3846 | / | / |
| **0.25** | SC | / | 0.62229 | 0.68179 |
|  | AA | 0.38423 | / | / |
| **0.5** | SC | / | 0.56169 | 0.62669 |
|  | AA | 0.46932 | / | / |
| **1.25** | SC | / | 0.5172 | 0.61516 |
|  | AA | 0.43135 | / | / |
| **2.5** | SC | / | 0.46597 | 0.5707 |
|  | AA | 0.40266 | / | / |

**Table S8. Summary of Results From Cholesterol Interference Study**

| **Cholesterol Concentration in Whole Blood, mg/mL** | **Sample** | **A-line data** | **S-line data** | **C-line data** |
| --- | --- | --- | --- | --- |
| **0** | SC | / | 0.56222 | 0.63548 |
|  | AA | 0.57409 | / | / |
| **0.2** | SC | / | 0.55836 | 0.64971 |
|  | AA | 0.55791 | / | / |
| **1** | SC | / | 0.57781 | 0.63676 |
|  | AA | 0.57439 | / | / |
| **2** | SC | / | 0.56893 | 0.63453 |
|  | AA | 0.58812 | / | / |
| **4** | SC | / | 0.5741 | 0.6287 |
|  | AA | 0.59567 | / | / |

**Table S9. Summary of Results From Reading Time Study**

| **Time** | **Sample** | **A-line data** | | | **S-line data** | | | **C-line data** | | |
| --- | --- | --- | --- | --- | --- | --- | --- | --- | --- | --- |
|  |  | A-mean | A-std | A-CV | S-mean | S-std | S-CV | C-mean | C-std | C-CV |
| **5 min** | SC | / | / | / | 0.53318 | 0.0047 | 0.9% | 0.6491 | 0.01924 | 3.0% |
|  | AA | 0.46334 | 0.023 | 5.0% | / | / | / | / | / | / |
| **7 min** | SC | / | / | / | 0.54794 | 0.0089 | 1.6% | 0.6447 | 0.01886 | 2.9% |
|  | AA | 0.45679 | 0.0177 | 3.9% | / | / | / | / | / | / |
| **9min** | SC | / | / | / | 0.54437 | 0.0176 | 3.2% | 0.6406 | 0.01782 | 2.8% |
|  | AA | 0.46527 | 0.0199 | 4.3% | / | / | / | / | / | / |
| **10 min** | SC | / | / | / | 0.58151 | 0.0109 | 1.9% | 0.7013 | 0.00315 | 0.4% |
|  | AA | 0.44283 | 0.003 | 0.7% | / | / | / | / | / | / |
| **25 min** | SC | / | / | / | 0.58254 | 0.0029 | 0.5% | 0.6895 | 0.00806 | 1.2% |
|  | AA | 0.45237 | 0.004 | 0.9% | / | / | / | / | / | / |
| **40 min** | SC | / | / | / | 0.55761 | 0.0167 | 3.0% | 0.6237 | 0.01358 | 2.2% |
|  | AA | 0.46176 | 0.0053 | 1.1% | / | / | / | / | / | / |
| **55 min** | SC | / | / | / | 0.51949 | 0.0273 | 5.3% | 0.5856 | 0.03202 | 5.5% |
|  | AA | 0.41944 | 0.0115 | 2.7% | / | / | / | / | / | / |
| **1h 10 min** | SC | / | / | / | 0.48632 | 0.0166 | 3.4% | 0.5468 | 0.02171 | 4.0% |
|  | AA | 0.42173 | 0.0077 | 1.8% | / | / | / | / | / | / |
| **2h 10 min** | SC | / | / | / | 0.49596 | 0.0065 | 1.3% | 0.5515 | 0.00774 | 1.4% |
|  | AA | 0.40301 | 0.0021 | 0.5% | / | / | / | / | / | / |
| **3 h 10 min** | SC | / | / | / | 0.50022 | 0.0044 | 0.9% | 0.5492 | 0.02001 | 3.6% |
|  | AA | 0.40498 | 0.007 | 1.7% | / | / | / | / | / | / |
| **4 h 10 min** | SC | / | / | / | 0.47873 | 0.013 | 2.7% | 0.5445 | 0.01459 | 2.7% |
|  | AA | 0.41454 | 0.0083 | 2.0% | / | / | / | / | / | / |
| **5 h 10 min** | SC | / | / | / | 0.50373 | 0.0098 | 1.9% | 0.5653 | 0.00706 | 1.2% |
|  | AA | 0.40967 | 0.008 | 2.0% | / | / | / | / | / | / |
| **6 h 10 min** | SC | / | / | / | 0.48678 | 0.009 | 1.8% | 0.5501 | 0.00316 | 0.6% |
|  | AA | 0.42613 | 0.0061 | 1.4% | / | / | / | / | / | / |
| **7 h 10 min** | SC | / | / | / | 0.48053 | 0.0067 | 1.4% | 0.5462 | 0.00377 | 0.7% |
|  | AA | 0.41593 | 0.0107 | 2.6% | / | / | / | / | / | / |
| **24 h** | SC | / | / | / | 0.40084 | 0.0079 | 2.0% | 0.5058 | 0.00758 | 1.5% |
|  | AA | 0.36086 | 0.0155 | 4.3% | / | / | / | / | / | / |

(/: indicates that the signal was below the cutoff)

**Table S10. Summary of Results From Visual Detection Study**

| Sample Genotype | Lines observed after 2 minutes | | |
| --- | --- | --- | --- |
|  | Technician 1 | Technician 2 | Technician 3 |
| Hb AA | A | A | A |
| Hb AA | A | A | A |
| Hb AS | AS | AS | AS |
| Hb AS | AS | AS | AS |
| Hb SC | SC | SC | SC |
| Hb SC | SC | SC | SC |
| Hb SS | SS | SS | SS |
| Hb SS | SS | SS | SS |
